# Traumatic high flow priapism in a child — the role of Colour Doppler Ultrasound and gelfoam embolotherapy for effective management

**DOI:** 10.1186/s42155-026-00730-7

**Published:** 2026-07-08

**Authors:** Sajal Patel, Richard Jenkins, Massimo Garriboli, Athanasios Diamantopoulos, Claire Lloyd, Narayan Karunanithy

**Affiliations:** 1 Interventional Radiology Department, Guy’s and St. Thomas’ NHS Foundation Trust, St. Thomas’ Hospital, 1St Floor, Lambeth Wing, Westminster Bridge Road, London, SE1 7EH UK; 2 Paediatric Radiology, Evelina London Children’s Hospital, London, UK; 3 Paediatric Urology, Evelina London Children’s Hospital, London, UK; 4 Stem Cells & Regenerative Medicine Section, Developmental Biology & Cancer Programme, UCL Institute of Child Health, London, UK; 5 School of Biomedical Engineering & Imaging Sciences, Faculty of Life Sciences & Medicine, King’s College London, London, UK

**Keywords:** Colour Doppler Ultrasound, Priapism, Arteriocavernous fistula, Embolisation

## Abstract

Post-traumatic priapism in the paediatric population poses a diagnostic and therapeutic challenge. Cavernous blood sampling is not well tolerated, and diagnostic uncertainty hinders management decisions. We present the use of Colour Doppler Ultrasound (CDU) to provide a definitive diagnosis of high-flow priapism and identification of an embolisation target which was then successfully embolised with gelfoam slurry with no clinical recurrence or complications at 6 months. Penile CDU is a reliable tool to diagnose arteriocavernous fistulae in children. Although conservative management remains first choice, safe and effective treatment of high-flow priapism in children can be achieved with gelfoam embolisation and identification of an embolisation target can aid the decision-making process.

## Introduction

High-flow priapism (HFP) is a relatively rare clinical condition, especially in comparison to low-flow or ischaemic priapism. The majority of HFP cases are secondary to trauma resulting in an arteriocavernous fistula or pseudoaneurysm of the cavernosal arteries causing an influx of arterial blood that exceeds venous drainage capacity [1]. The penile tumescence is typically less pronounced than in ischaemic priapism and is usually painless. Treatment also differs significantly between the two aetiologies and it is imperative that a conclusive diagnosis of HFP be achieved to reduce the risk of erectile dysfunction (ED). A cavernous blood sample demonstrating a high oxygen tension is diagnostic of HFP; however, in the paediatric population, this investigation is likely to be poorly tolerated and may require anaesthetic support. An ultrasound examination may yield evidence of trauma and Colour Doppler Ultrasound (CDU) may pinpoint the traumatic fistula or pseudoaneurysm and the parent vessel.

Treatment options broadly include conservative measures, embolisation and surgical repair. Surgical repair has been associated with a risk of ED [2] and while embolisation in the treatment of priapism has been documented since 1977 [3], there is no consensus regarding the embolic agent of choice.

Here we present a case of traumatic HFP, diagnosed with CDU and successfully treated with embolisation.

## Case report

A 9-year-old patient presented with priapism following a blunt perineal injury sustained from a bicycle handlebar 8 days prior. The penis was fully erect with adjacent bruising. Detumescence was seen on applying perineal pressure.

**Fig. 1:**
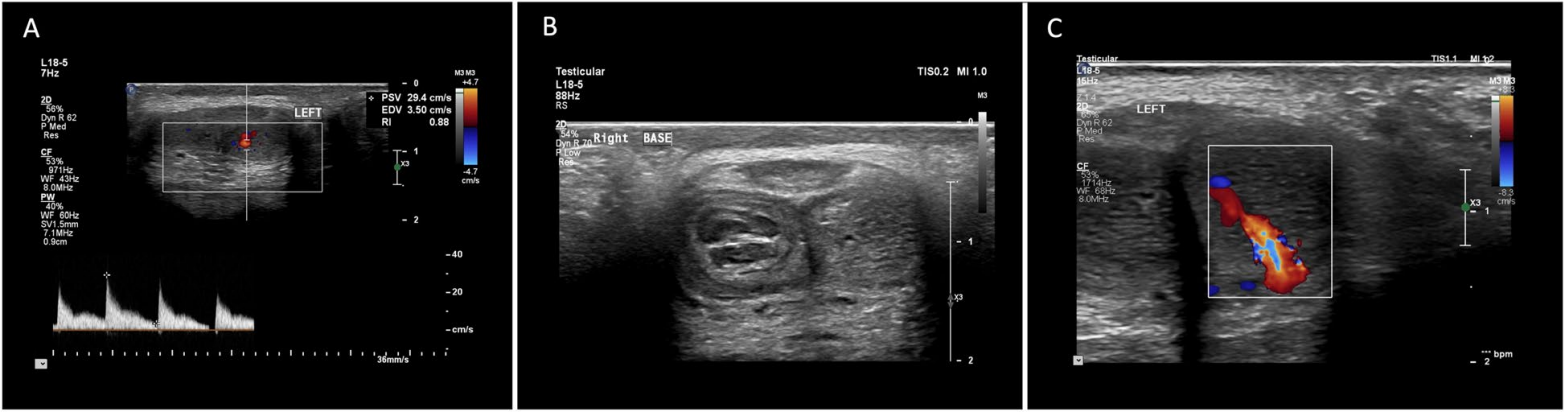
Diagnostic Colour Doppler Ultrasound (CDU) at presentation. **A** CDU of the left cavernosal artery demonstrates a high-flow, low resistance waveform with a peak systolic velocity of 29 cm/sec. **B** Haematoma seen in the right corpus cavernosum on B-mode imaging. **C** Fistula demonstrated in the left corpus cavernosum as a jet of turbulent flow

Penile CDU (see Fig. 1) was performed and demonstrated bilateral hypoechoic structures in the corpora cavernosa in keeping with haematomas between the base and midshaft. Turbulent colour flow was seen in some parts of these haematomas whereas other areas appeared avascular. The cavernosal arteries appeared to communicate with the areas of turbulent flow. A high-flow, low-resistance waveform was demonstrated in the left cavernosal artery consistent with non-ischaemic priapism. A fistula was clearly visualised on Colour Doppler imaging as a jet of turbulent flow on the left. Findings were consistent with bilateral haematomas with arteriocavernosal fistula.

Following a multidisciplinary team discussion, the options of conservative management versus embolisation therapy and the risks involved in both were outlined in a detailed discussion with the parent. The risks of not treating (permanent ED and corporal fibrosis) were carefully balanced against the risks of treatment (ED and ischaemia). The direct target for embolisation provided by the results of the CDU was also considered. The decision was made to proceed to embolisation.

**Fig. 2:**
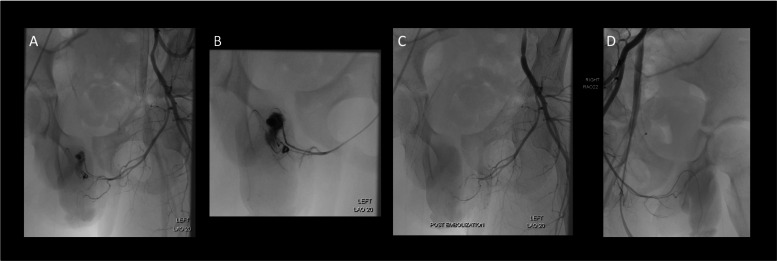
Angiographic images. **A** Angiogram from the left internal iliac artery demonstrating an arteriocavernosal fistula arising from the cavernosal artery. **B** Angiogram following superselective cannulation of the left cavernosal artery demonstrating the fistula, feeding branches and confirming a safe embolisation starting point. **C** Post-embolisation image demonstrates preservation of surrounding branches. **D** Angiogram from the right internal iliac artery rules out right-sided arteriocavernous fistula and filling of the left-sided fistula from the right

Under general anaesthesia, the right common femoral artery was accessed with ultrasound guidance and access secured with a 4Fr sheath. An angiogram was performed from the aortic bifurcation followed by cannulation of the left internal pudendal artery. The angiogram revealed an arteriocavernosal fistula arising from the left cavernosal artery (see Fig. 2) with early opacification of the left corpus cavernosum. Following the administration of 400 mcg of isosorbide mononitrate to prevent vasospasm, the left cavernosal artery was superselectively cannulated with a 2.4Fr Progreat™ microcatheter (Terumo Corporation, Tokyo, Japan). Embolisation with gelfoam slurry resulted in obliteration of the fistula while preserving adjacent branches. An angiogram from the right internal pudendal artery demonstrated normal appearances of the right cavernosal and dorsal penile arteries. Haemostasis was achieved with manual compression.

**Fig. 3:**
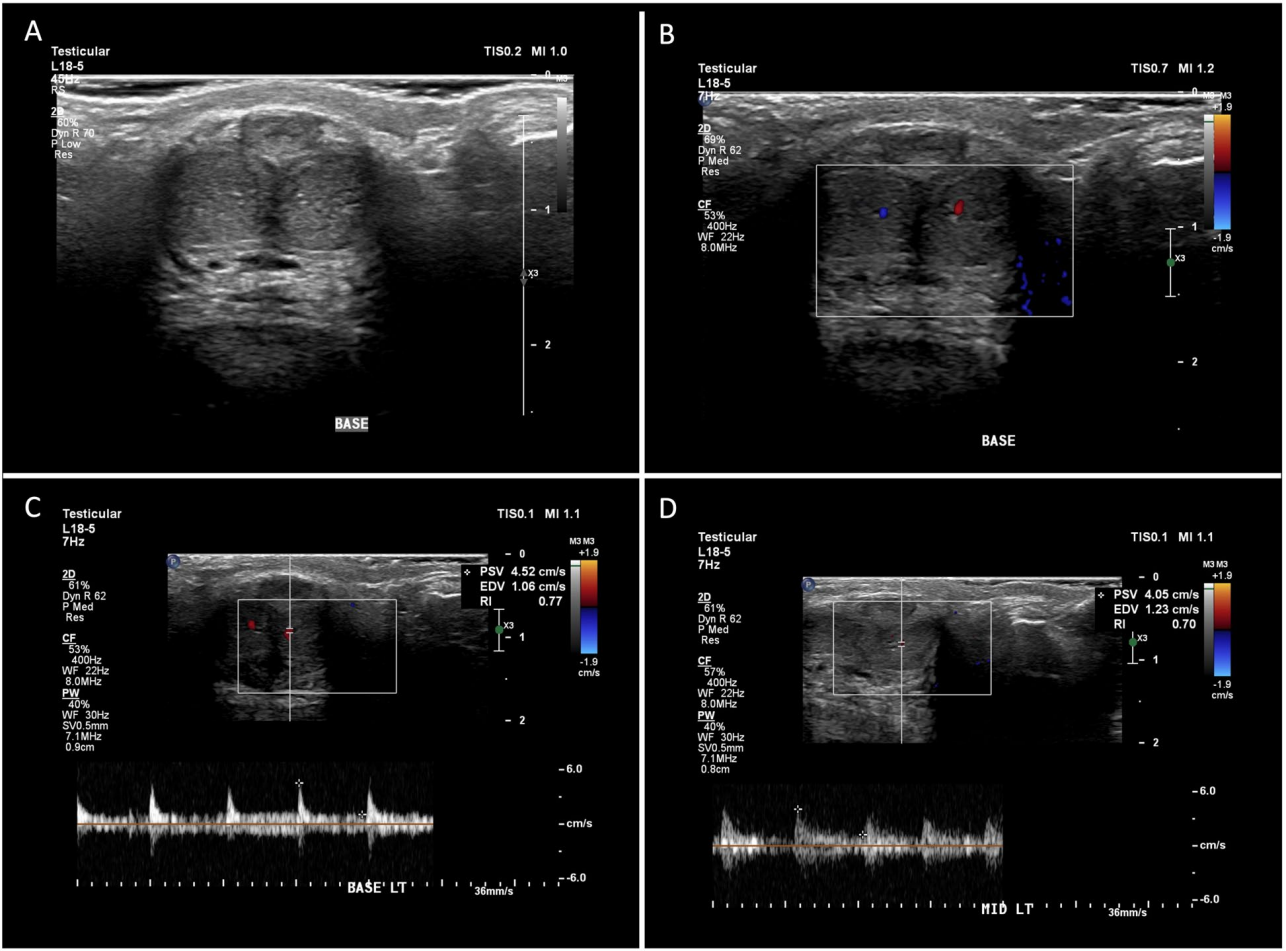
Follow-up ultrasound. **A** B-mode image demonstrating resolution of cavernosal haematoma. **B** No residual left cavernosal fistula. **C**, **D** Restoration of the normal low-flow, low resistance waveform in the proximal and mid left cavernosal arteries with a peak systolic velocity of 6 cm/sec

Post-operatively, detumescence was seen the following day; there were no reports of pain or fever and the patient was voiding well. An ultrasound at 5 weeks demonstrated no residual fistula and no fibrosis (see Fig. 3). There was complete resolution of the previously seen hypoechoic structures and normal waveforms throughout both cavernosal arteries. The patient remains free of recurrent symptoms or complications at 6 months.

## Discussion

Persistent high-flow priapism poses a therapeutic challenge especially in the paediatric population. There is no current consensus on conservative management versus embolisation. Conservative management may result in symptom resolution; however, a CDU that clearly demonstrates a fistula or pseudoaneurysm can aid decision making and prompt definitive treatment. CDU features of HFP (Fig. 1) and comparison with low-flow (ischaemic) priapism that typically demonstrates high resistance waveform have been previously described [4, 5]. Furthermore, the use of CDU intraoperatively may reduce the amount of radiation required during an embolisation procedure as well as aid in the preservation of intact vessels in variant anatomy, thereby reducing the risk of ED [6]. Whilst the CDU in our case clearly demonstrated an arteriocavernous fistula. A high index of suspicion should be maintained of this finding in a strongly symptomatic post-traumatic child where the CDU is negative. Angiographic confirmation of the arteriocavernous fistula can be justified in such cases.

Our case demonstrates a scenario where there is no clearly optimal management option, in this case, a CDU which clearly demonstrated an arteriocavernous fistula provided a target for embolisation and the possibility for definitive treatment. CDU also provides a non-invasive follow-up modality which can detect recurrence, vessel recanalisation, collateral pathway formation [7] and complications such as fibrosis.

The choice of embolic agent reported in the literature varies widely. The use of a resorbable agent such as gelfoam is beneficial in that delayed recanalisation of the occluded vessels is possible reducing the risk of ischaemic ED. This may however also result in recurrence of the fistula and occasionally repeat treatments are required [8]. Our patient has remained well postoperatively with no clinical evidence of recurrence at 6 months. Percutaneous ultrasound-guided thrombin injection is a minimally invasive alternative to transarterial embolisation. No direct comparisons of outcomes exist currently [9]. Whilst gelfoam is preferred as the first-line embolic agent, recurrent HFP may necessitate the use of permanent embolic agents like microcoils, polyvinyl alcohol and glue [10].

Small calibre paediatric vessels are prone to vasospasm and hence the authors recommend the prophylactic use of isosorbide dinitrate as is routine in other practices like radial artery access. No published guidance on dose regimes exists; however, 10 mcg/kg up to 500 mcg is routinely used.

## Conclusion

Penile CDU is a reliable tool to diagnose arteriocavernous fistulae in children. Although conservative management remains first choice, safe and effective therapy of high-flow priapism in children can be achieved with gelfoam embolisation and identification of an embolisation target can aid the decision-making process.

## Data Availability

Anonymised imaging is available from the corresponding author on reasonable request.

## Abbreviations

CDU: Colour Doppler Ultrasound
HFP: High-flow priapism
ED: Erectile dysfunction
